# Frontal facial proportions of 12-year-old southern Chinese: a photogrammetric study

**DOI:** 10.1186/s13005-015-0084-7

**Published:** 2015-08-14

**Authors:** Charles Yat Cheong Yeung, Colman Patrick McGrath, Ricky Wing Kit Wong, Erik Urban Oskar Hägg, John Lo, Yanqi Yang

**Affiliations:** Department of Paediatric Dentistry and Orthodontics, Faculty of Dentistry, The University of Hong Kong, Hong Kong SAR, China; Department of Periodontology and Public Health, Faculty of Dentistry, The University of Hong Kong, Hong Kong SAR, China; Department of Paediatric Dentistry and Orthodontics, Faculty of Dentistry, The University of Hong Kong, Hong Kong SAR, China; Department of Paediatric Dentistry and Orthodontics, Faculty of Dentistry, The University of Hong Kong, Hong Kong SAR, China; Department of Oral and Maxillofacial Surgery, Faculty of Dentistry, The University of Hong Kong, Hong Kong SAR, China; Department of Paediatric Dentistry and Orthodontics, Faculty of Dentistry, The University of Hong Kong, Hong Kong SAR, China

**Keywords:** Facial proportions, Southern Chinese, Photogrammetry, Population norm, Facial attractiveness, Diagnosis, Treatment outcome evaluation, Orthodontics, Orthognathic surgery, Plastic surgery, Cosmetic surgery

## Abstract

This study aimed to establish norm values for facial proportion indices among 12-year-old southern Chinese children, to determine lower facial proportion, and to identify gender differences in facial proportions.

A random population sample of 514 children was recruited. Fifteen facial landmarks were plotted with ImageJ (V1.45) on standardized photos and 22 Facial proportion index values were obtained. Gender differences were analyzed by 2-sample t-test with 95 % confidence interval. Repeated measurements were conducted on approximately 10 % of the cases.

The rate of adopted subjects was 52.5 % (270/514). Intraclass correlation coefficient values (ICC) for intra- examiner reliability were >0.87. Population facial proportion index values were derived. Gender differences in 11 of the facial proportion indices were evident (*P* < 0.05).

Upper face-face height (N- Sto/ N- Gn), vermilion height (Ls-Sto/Sto-Li), upper face height-biocular width (N-Sto/ExR-ExL) and nose -face height (N-Sn/N-Gn) indices were found to be larger among girls (*P* < 0.01). Males had larger lower face-face height (Sn -Gn/ N-Gn), mandibulo-face height (Sto-Gn/N-Gn), mandibulo-upper face height (Sto-Gn/N-Sto), nasal (AlR-AlL/N-Sn), upper lip height-mouth width (Sn-Sto/ChR-ChL), upper lip-upper face height (Sn-Sto/N-Sto) and upper lip-nose height (Sn-Sto/N-Sn) indices (*P* < 0.05).

Population norm of facial proportion indices for 12-year-old Southern Chinese were derived and mean lower facial proportion were obtained. Sexual dimorphism is apparent.

## Introduction

Facial attractiveness has been a subject of interest since the beginning of recorded history. Bashour reviewed the historical and current literatures and concluded with four important cues that emerge as being the most important determinants of facial attractiveness [1]. They are: (i) averageness, (ii) sexual dimorphism, (iii) youthfulness, and (iv) symmetry. Averageness is regarded as one of the most important factors and supported by various studies [2–6].

Facial attractiveness has long been of central concern to orthodontic and surgical care given that treatments are capable of changing facial appearance and thereby improve facial attractiveness [7]. It is therefore important to establish population norms to address the averageness cue, and provide insight on sexual dimorphism and youthfulness. Symmetry can be assessed clinically without the need of a norm.

Farkas suggested the use of facial proportion indices to assess aesthetics relating to facial proportions in different facial types [8]. Edler quantified facial attractiveness after orthognathic surgery and found, the greater the improvement in facial proportion indices, the better the aesthetic result as judged by orthodontists and maxillofacial surgeons [6]. These post surgical indices correlated closely to Farkas’ findings. Facial proportion is, therefore, important in both clinical diagnosis and treatment outcome evaluation.

Photogrammetry is increasingly being employed to assess facial characteristics [6, 9–14]. It is reported to be valid for many measurements [15, 16], reliable [6, 10–13, 16, 17] and is a practical approach to clinical analyses and comparison [6, 14, 17].

While facial proportion norms are well-established for Caucasian populations [9], it remains paucity for southern Chinese.

The aims of this study were:

I. To provide a database of norm of facial proportion indices for 12-year-old southern Chinese for surgical and orthodontic diagnosis.
II. To determine sexual dimorphism in facial proportions.
III. To determine the lower facial proportion

## Materials and method

### Sample

This epidemiological study was conducted in Hong Kong SAR, China among 12-year-old children. Ethical approval was obtained by the local IRB committee (UW 09–453). Ten percent of all secondary schools in Hong Kong SAR were randomly selected and children within each selected school were invited to participate. Written informed consent was obtained from parents and children provided their ascent. A sample of 514 (259 males, 255 females) 12-year-old children was recruited. That was approximately 10 % of the Chinese birth cohort since all 12-year-old children in Hong Kong, spread over all secondary schools, are included in the cohort.

### Photographic set up

A scale backdrop of 1 cm increments with a plumbline was set up for a camera-subject distance of around 170 cm. The camera used was Canon EOS 400D (Canon, Shimomaruko, Ohta-ku, Tokyo, Japan) with Canon EF-S 60 mm f/2.8 Macro USM Lens and Canon MR-14EX TTL Macro Ring Lite Flash. Subjects were first instructed to a stand with their eyes looking forward to a vertically standing mirror on the side for a natural head posture and then turn their whole body 90° to face the camera with the lip relax. Glasses or other accessories which may obstruct the face were taken away beforehand. The photo was then taken in natural head posture [18].

### Selection of landmarks and proportion indices: (Fig. 1)

**Fig. 1:**
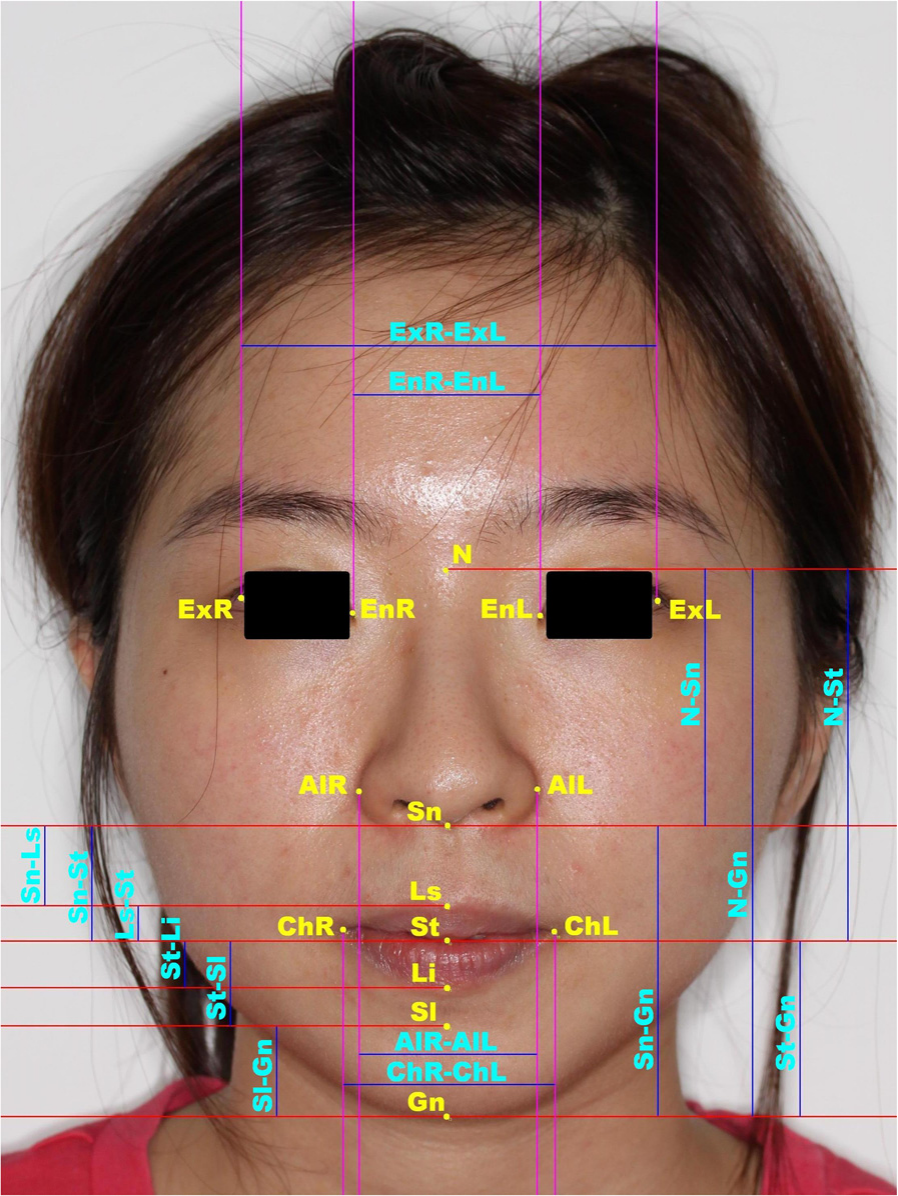
Landmarks and proportional indices identified and measured. N : Nasion; Ex (R,L) : Exocanthion (*Right, Left*); En (R,L) : Endocanthion (*Right, Left*); Al (R,L) : Alare (*Right, Left*); Sn : Subnasale; Ls : Labiale superius; Sto : Stomion; Li : Labiale inferius; Ch (R,L) : Cheilion (*Right*, *Left*); Sl : Sublabiale; Gn : Gnathion. N.B. The subject of this photograph is not a subject in the study, the photograph is for illustration purpose and consent was obtained

Fifteen landmarks (Fig. 1) as employed by Farkas and Munro [8] were considered; based on key variables considered by Bishara [10–12] (1) visibility in most frontal photographs (2) reliable identification (3) minimally affected by the subject’s grooming, and (4) involved in the measurement of proportional indices of interest. Twenty-two proportion indices (Table 1) employed by Farkas and Munro [8] were selected to be investigated basing on key variables considered by Bishara [10–12], and Edler [6] (1) Measurable on a frontal photographs; (2) reliability; and (3) be potentially changed by the effects of orthodontics and/or orthognathic surgery.

**Table 1:** Descriptions of Facial Proportion Indices abd their respective intraclass correlation coefficients of intra examiner reliability

Index	Description	Intra-examiner reliability (ICC)
Upper face-face height index	N- Sto/ N- Gn ×100	1
Lower face-face height index	Sn -Gn/ N-Gn ×100	0.99
Mandibulo-face height index	Sto-Gn/N-Gn ×100	1
Mandibulo-upper face height index	Sto-Gn/N-Sto ×100	1
Mandibulo-lower face height index	Sto-Gn/Sn-Gn ×100	0.99
Nasal index	AlR-AlL/N-Sn ×100	0.88
Upper lip height-mouth width index	Sn-Sto/ChR-ChL ×100	0.98
Cutaneous-total upper lip height index	Sn-Ls/Sn-Sto ×100	0.95
Vermilion-total upper lip height index	Ls-Sto/Sn-Sto ×100	0.95
Vermilion-cutaneous upper lip height index	Ls-Sto/Sn-Ls ×100	0.95
Vermilion height index	Ls-Sto/Sto-Li ×100	0.96
Chin-mandible height index	Sl-Gn/Sto-Gn ×100	0.9
Upper face height-biocular width index	N-Sto/ExR-ExL ×100	0.99
Intercanthal-nasal width index	EnR-EnL/AlR-AlL ×100	0.99
Nose-face height index	N-Sn/N-Gn ×100	0.99
Nose-mouth width index	AlR-AlL/ChR-ChL ×100	0.98
Upper lip-upper face height index	Sn-Sto/N-Sto ×100	0.98
Upper lip-mandible height index	Sn-Sto/Sto-Gn ×100	0.99
Upper lip-nose height index	Sn-Sto/N-Sn ×100	0.97
Lower lip-face height index	Sto-Sl/Sn-Gn ×100	0.89
Lower lip-mandible height index	Sto-Sl/Sto-Gn ×100	0.9
Lower lip-chin height index	Sto-Sl/Sl-Gn ×100	0.91

### Selection of photos

Photos were inspected for their quality and usability in identification of landmarks and validity in measurement of the proportion indices. Photos were excluded if: (1) landmarks were obscured; (2) head tilted up or down significantly; (3) head turned left or right by assessing symmetrical structures; (4) out focused photo; (5) subject wearing glasses; (6) subject showing lip strain or obviously opened mouth; (7) subject smiled; (8) patient partly or completely closed their eyes; and (9) subject previously or currently having orthodontic treatment.

### Digitalization of photos

The selected photos were cropped to show the head only. A tangent at superior palpebral sulci was used to determine the vertical level of Nasion [8, 19]. Landmarks were located with ImageJ (V.1.45) (USA National Institutes of Health) and position of each landmark was recorded as a set of X-Y pixel coordinates by the same trained operator. The proportion indices were generated by Microsoft Excel® with the formulas in Table 1.

### Statistical analysis

For each facial proportion index, descriptive statistics of mean, standard deviation and range (maximum, minimum) were generated by Statistical Product and Service Solutions (V20) (IBM Corporation, New York, USA). Two sample T- test (with 95 % confidence interval provided) were used to identify any gender difference. The intraclass correlation coefficients [20] were calculated by SPSS (V.19) to assess for intra-examiner reliability, among approximately 10 % of randomly selected subjects that were re-analyzed and compared to original assessments.

## Results

Out of 514 subjects, 53 % (270) were included in the analysis, among that, 51 % (137) were female. 47 % (244) were excluded according to the exclusion criteria (as described above). Intra-examiner reliability is presented in Table 1. The intraclass correlation coefficients were all above 0.87 for intra examiner reliability, indicating very good/excellent reliability [20].

Facial proportion index norm values are presented in Table 2. Greatest variance were observed in vermilion cutaneous-upper lip height (Males: 29.58–145.59, Females: 28.81–124.32), vermilion height (Males: 55.26–120.75, Females: 44.59–175.00) and lower lip-chin height (Males: 36.25–135.63, Females: 25.44–131.40) indices. Lowest variances were observed in upper face-face height (Males: 56.73–70.79, Females: 58.35–70.19), lower face-face height (Males: 50.34–62.69, Females: 48.06–60.95) and mandibulo-lower face height (Males: 57.98–72.07, Females: 58.64–70.64) indices. From lower face-face height index, the proportion of lower face was 56 % of total face height. The proportion of upper lip height (Sn-Sto), lower lip height (Sto-Sl) and chin height (Sl-Gn) were found to be 35.12, 27.33 and 37.55 % respectively.

**Table 2:** Mean, standard deviation, maximum, minimum and p-values of statistical tests (2 sample t-test) of facial proportion indices

	Male (*n* = 133)				Female (*n* = 137)				
	Mean	SD	Min.	Max.	Mean	SD	Min.	Max.	*P*-value
Upper face-face height index	63.27	2.58	56.73	70.79	63.92	2.36	58.35	70.19	0.031*
Lower face-face height index	56.75	2.44	50.34	62.69	55.25	2.59	48.06	60.95	0.000***
Mandibulo-face height index	36.73	2.58	29.21	43.27	36.08	2.36	29.81	41.65	0.031*
Mandibulo-upper face height index	58.33	6.54	41.25	76.26	56.66	5.80	42.47	71.37	0.028*
Mandibulo-lower face height index	64.69	2.83	57.98	72.07	65.29	2.63	58.64	70.64	0.075
Nasal index	77.12	5.69	64.32	93.83	73.99	6.54	60.70	93.94	0.000***
Upper lip height-mouth width index	52.02	6.47	37.09	71.21	49.36	6.21	36.46	67.05	0.001**
Cutaneous-total upper lip height index	62.16	6.84	40.72	77.17	61.99	6.82	44.58	77.63	0.838
Vermilion-total upper lip height index	37.84	6.84	22.83	59.28	38.01	6.82	22.37	55.42	0.837
Vermilion-cutaneous upper lip height index	63.00	19.83	29.58	145.59	63.30	18.37	28.81	124.32	0.898
Vermilion height index	76.61	12.08	55.26	120.75	82.21	19.80	44.59	175.00	0.005**
Chin-mandible height index	58.12	5.57	42.44	73.39	58.78	6.03	43.22	79.72	0.352
Upper face height-biocular width index	85.33	5.01	73.41	98.37	86.92	4.54	74.69	98.24	0.007**
Intercanthal-nasal width index	95.22	6.70	79.48	112.82	95.37	7.75	77.32	113.96	0.871
Nose -face height index	43.25	2.44	37.31	49.66	44.75	2.59	39.05	51.94	0.000***
Nose-mouth width index	86.22	7.03	68.69	109.87	84.70	7.02	70.98	109.49	0.075
Upper lip-upper face height index	31.65	2.31	25.60	37.19	30.02	2.55	24.28	35.99	0.000***
Upper lip-mandible height index	54.87	6.76	38.75	72.48	53.42	6.22	41.57	70.55	0.067
Upper lip-nose height index	46.46	4.96	34.41	59.21	43.08	5.26	32.07	56.23	0.000***
Lower lip-face height index	27.05	3.43	17.07	35.81	26.87	3.80	12.91	36.22	0.693
Lower lip-mandible height index	41.88	5.57	26.61	57.56	41.22	6.03	20.28	56.79	0.352
Lower lip-chin height index	73.66	17.05	36.25	135.63	71.94	18.03	25.44	131.40	0.421

*P-value<0.05, **P-value<0.01, ***P-value<0.001

Gender differences in 11 of the 22 facial proportion indices assessments were apparent. For females, upper face-face height (*P* < 0.05), vermilion height (*P* < 0.001), upper face height-biocular width (*P* < 0.01) and nose -face height (*P* < 0.001) indices were larger. In contrast, lower face-face height (*P* < 0.001) mandibulo-face height (*P* < 0.05), mandibulo-upper face height (*P* < 0.05), nasal (*P* < 0.001), upper lip height-mouth width (*P* = 0.001), upper lip-upper face height (*P* < 0.001) and upper lip-nose height (*P* < 0.001) indices were larger in males. The index with the largest mean percentage difference (7.56 %) between genders was upper lip-nose height index.

## Discussion

This study was conducted on a random population sample of 12-year-old children, as opposed to small, non-random, convenient samples as described in the majority of photogrammetric studies published to date [21–23]. With the rate of adopted subjects of 53 %, there are a number of factors to account for the loss of samples including less than ideal cooperation from 12 years old children, time constraint and past or current orthodontic treatment. This reflects the difficulties in performing population-wide photogrammetric studies under non-clinical/outreach settings in the community. Nevertheless, the sample size was sufficient to provide populations norms and to discover gender differences and it is one of the largest samples for photogrammetric study with random population sample and largest range of assessment of proportion indices.

A smaller local study analyzed facial profiles with photogrammetry on only 82 12-year-old southern Chinese with just five proportion indices [21]. Findings were consistent with the present study, with mean differences less than 1 standard deviation for four indices: lower-face-face height index, mandibulo-lower face height, intercanthal-nasal width and lower lip-face height indices. The only inconsistency was reported for the lower lip-face height index, which they reported a statistically significant gender difference. In comparison with northern America Caucasian population [8], the only index that differed by more than 2 standard deviation is the nose-mouth width index for both males and females. This indicates that southern Chinese have a relatively wider nose (AlR-AlL) or narrower mouth (ChR-ChL) compared to Caucasians. The nasal index for males, upper face height-biocular width index and upper-lip-mandible height index for females were larger in southern Chinese by almost 2 standard deviations. The reverse was found for the female mandibulo-upper face height index.

Of a particular importance to orthodontics and orthognathic surgery is the lower face proportion. The lower face height in our study was found to be 56.7 %(male) and 55.3 %(female) of the total face height corresponding well to lateral cephalometric study [24] (M:56.5 %, F:55.7 %), photogrammetric study [25] in Nigerian adults (M:58.15 %, F:56.97 %) and anthropotmetric study [8] on 12-year-old Caucasian children (M:59.7 %, F:59.5 %). Regarding the proportions of the lower third of the face, Renaissance artist Francesca [8] suggested that the lower lip and chin should make up two thirds of the lower one third of face and lower lip and chin should have the same proportion, this is widely adopted in orthodontics and surgery text. Farkas had found proportionality from anthropometric study [8], which is 31.2, 26.2 and 42.6 % for Sn-Sto, Sto-Sl and Sl-Gn. In our study, the proportion was 35.1, 27.3 and 37.6 % respectively.

Farkas [8], Song [26] and Bao [27] reported that there gender difference in facial dimension and proportions but the average differences were small. The results from this study generally supports Farkas’ conclusion. In this study, 11 (50 %) out of 22 facial proportion indices showed significant gender differences. All except 4 of the indices had a percentage difference of less than 5 %. They are upper lip-nose height (7.6 %), vermilion height (7.0 %), upper lip-upper face height (5.3 %) and upper lip height-mouth width (5.3 %) indices.

The norm facial proportion indices obtained can be used for clinical assessment and comparison with same analysis and photogrammetric technique. Frontal photogrammetry was widely used to assess treatment change [6, 9–12], attractiveness [13, 28, 29], comparisons between different ethnic groups [13, 14, 30] and growth [10–12, 31] in additional to daily use for clinical diagnosis and treatment planning.

To conclude, the following were the key findings of this study.

I. Population norm of facial proportion indices are obtained from the mean values of this study and can serve as a reference to evaluate facial proportions in treatment planning and treatment outcome assessment using the same frontal photogrammetric analysis.
II. Gender differences in facial proportion were found in 11 indices. Lower face-face height index, mandibulo-face height index, mandibulo-upper face height index, nasal index, upper lip height-mouth width index, upper lip-upper face height index and upper lip-nose height index were significantly larger in males. The findings were opposite for upper face-face height index, vermilion-height index, upper face height-biocular width index and nose -face height index.
III. The lower face height is found to be 56.7 %(male) and 55.3 %(female) of the total face height and proportions of lower facial height were 35.1, 27.3 and 37.6 % for Sn-Sto, Sto-Sl and Sl-Gn respectively.
IV. Ethnic differences were evaluated by comparing with a North American Caucasian population, southern Chinese was found to have a relatively wider nose (AlR-AlL) or narrower mouth (ChR-ChL) compared to the Whites.
